# Heterozygous Genetic Variants in Autosomal Recessive Genes of the Leptin-Melanocortin Signalling Pathway Are Associated With the Development of Childhood Obesity

**DOI:** 10.3389/fendo.2022.832911

**Published:** 2022-04-29

**Authors:** Robert Šket, Primož Kotnik, Barbara Jenko Bizjan, Valentina Kocen, Matej Mlinarič, Tine Tesovnik, Maruša Debeljak, Tadej Battelino, Jernej Kovač

**Affiliations:** ^1^ Clinical Institute of Special Laboratory Diagnostics, University Children’s Hospital, University Medical Center Ljubljana (UMC), Ljubljana, Slovenia; ^2^ Department of Pediatrics Endocrinology, Diabetes and Metabolic Diseases, University Children’s Hospital, University Medical Center Ljubljana (UMC), Ljubljana, Slovenia; ^3^ Faculty of Medicine, University of Ljubljana, Ljubljana, Slovenia

**Keywords:** childhood obesity, leptin-melanocortin pathway, hyperphagia, next-generation sequencing, genetic screening

## Abstract

Monogenic obesity is a severe, genetically determined disorder that affects up to 1/1000 newborns. Recent reports on potential new therapeutics and innovative clinical approaches have highlighted the need for early identification of individuals with rare genetic variants that can alter the functioning of the leptin-melanocortin signalling pathway, in order to speed up clinical intervention and reduce the risk of chronic complications. Therefore, next-generation DNA sequencing of central genes in the leptin-melanocortin pathway was performed in 1508 children and adolescents with and without obesity, aged 2-19 years. The recruited cohort comprised approximately 5% of the national paediatric population with obesity. The model-estimated effect size of rare variants in the leptin-melanocortin signalling pathway on longitudinal weight gain between carriers and non-carriers was derived. In total, 21 (1.4%) participants had known disease-causing heterozygous variants (DCVs) in the genes under investigation, and 62 (4.1%) participants were carriers of rare variants of unknown clinical significance (VUS). The estimated frequency of potential genetic variants associated with obesity (including rare VUS) ranged between 1/150 (VUS and DCV) and 1/850 (DCV) and differed significantly between participants with and without obesity. On average, the variants identified would result in approximately 7.6 kg (7.0-12.9 kg at the 95th percentile of body weight) (girls) and 8.4 kg (8.2-14.4 kg) (boys) of additional weight gain in carriers at age 18 years compared with subjects without obesity. In conclusion, children with a genetic predisposition to obesity can be promptly identified and may account for more than 6% of obesity cases. Early identification of genetic variants in the *LEPR*, *PCSK1*, *POMC*, *MC3R* and *MC4R* genes could reduce the societal burden and improve the clinical management of early severe childhood obesity and its implementation should be further investigated.

## Introduction

Overweight and obesity are defined as the excessive accumulation of fat that poses a risk to health (1). It is associated with an increased risk of chronic diseases, comprising metabolic syndrome, cardiovascular disease, type II diabetes, dyslipidaemia and cancer, as well as adverse psychological consequences, manifested in weight stigma and low self-esteem (2–4). In 2019, 38.2 million children under 5 years and 340 million adolescents aged 5-19 years worldwide were overweight or obese according to the WHO child growth standards (BMI-for-age for obesity >3 SD above the median of the WHO standards for children under 5 years and >2 SD for children aged 5-19 years) (1, 5, 6). Heritability estimates range between 40% and 70% for BMI and indicate a relatively strong genetic predisposition to obesity (3). Most genetically determined obesity is of polygenic origin, in which the cumulative contribution of an obesogenic lifestyle and several genes with more subtle additive effects results in increased body weight (7–9).

**Figure 1:**
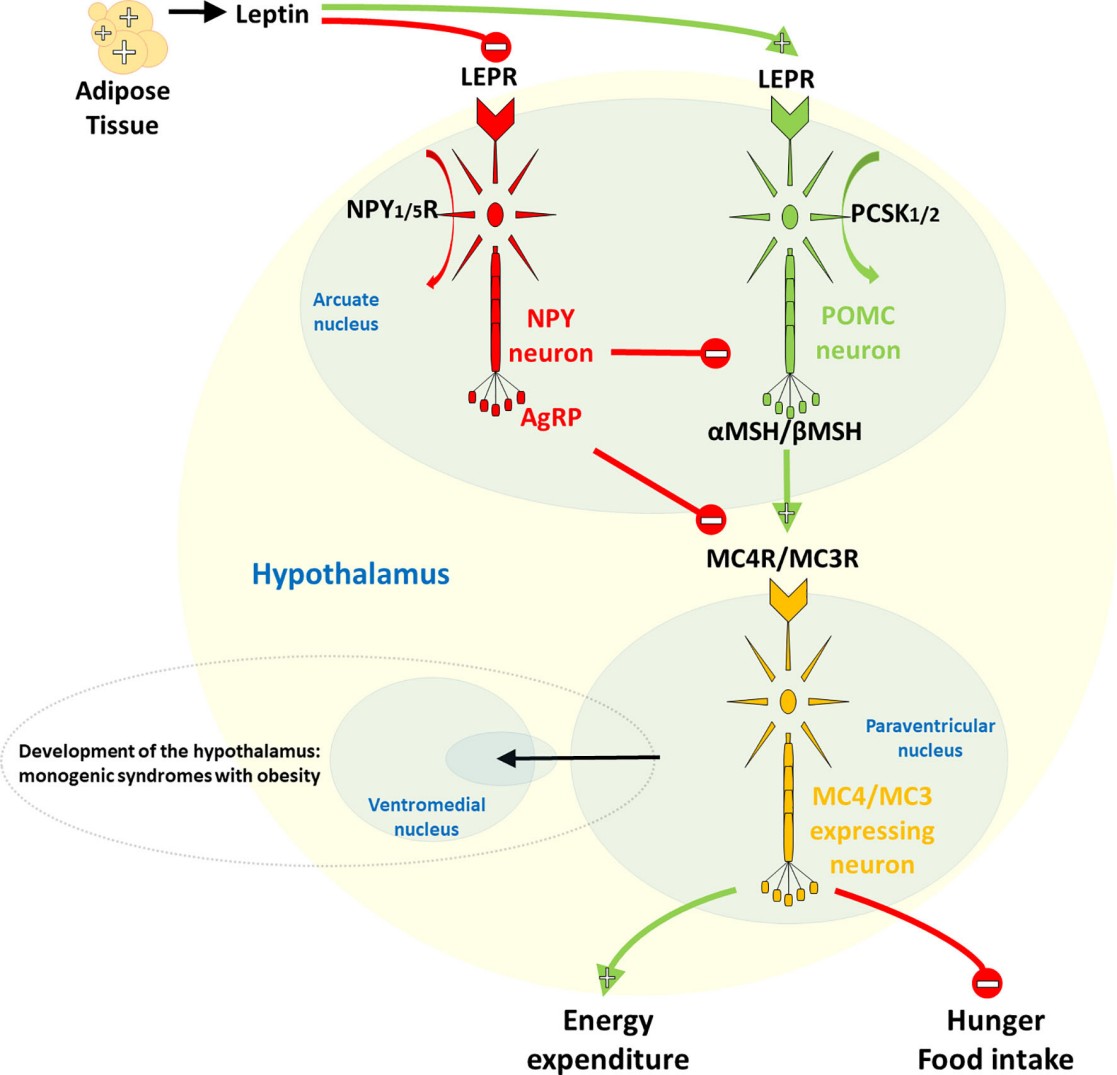
Hypothalamic leptin-melanocortin system. The first in the cascade is the anorexigenic hormone leptin, whose blood level corresponds to the mass of white adipose tissue and which acts as a leptin receptor (*LEPR*) agonist*. LEPR* is further linked to differential neuronal involvement of neuropeptide Y (*NPY*)/agouti-related neuropeptide (*AgRP*) and proopiomelanocortin (*POMC*), with no inhibition by *NPY*/*AgRP*, *POMC*, on the other hand, [via its proprotein convertase subtilisin/kexin type 1/2 (*PCSK1/2*) cleavage activity] stimulates the melanocortin-4 receptor (*MC4R*) and the melanocortin-3 receptor (*MC3R*), ultimately leading to increased energy expenditure and decreased satiety.

On the other hand, rare forms of obesity due to a single dysfunctional gene, due to small or large chromosomal deletions or single gene defects inherited in a Mendelian pattern, usually manifest in severe and early-onset obesity with very limited environmental influence on its development (10, 11). Monogenic obesity is present in 2-3% of obese children and adults, corresponding to a prevalence of ~1-2/1000 persons, and is most commonly caused by mutations in genes encoding the leptin-melanocortin pathway in the central nervous system (CNS) (**Figure 1**) (10, 12). First in the cascade of the leptin-melanocortin system is the anorexigenic hormone leptin, with blood-circulating levels corresponding to the mass of white adipose tissue and its function as a leptin receptor (*LEPR*) agonist. The *LEPR* gene is further associated with the different engagement of proopiomelanocortin (*POMC*) neurons and neuropeptide Y (*NPY*)/agouti-related neuropeptide (*AgRP*), with *POMC* [through proprotein convertase subtilisin/kexin type 1/2 (*PCSK1/2*) cleavage activity] stimulating and *NPY*/*AgRP* not inhibiting receptors melanocortin-4 receptor (*MC4R*) and melanocortin-3 receptor (*MC3R*) which, in the end, result in increased energy expenditure and reduced satiety. In addition to controlling the hedonic aspects of eating patterns and energy expenditure, endogenous ligands and receptors in this pathway also control glucose metabolism, blood pressure and heart rate. Although causative mutations are difficult to confirm, mutations in most genes of the leptin-melanocortin pathway that cause hyperphagia and severe obesity in humans and mice are, in particular, pathogenic mutations in the *MC4R* gene, which have been found in up to 5% of cases of early childhood obesity and up to 0.3% of the general population (7, 13).

Knowing an individual’s genetic susceptibility allows for a more accurate diagnosis of the type of obesity and the identification of targets for anti-obesity treatment. Short peptides, e.g. setmelanotide, are now in various phases of clinical trials for the treatment of patients with severe obesity due to rare genetic disorders in the leptin-melanocortin pathway (14–16). This suggests the potential benefit of molecular genetic screening to identify genetically determined obesity and the possibility of early behavioural intervention complemented by innovative pharmacological treatments (7, 16).

In this study, we first aim to determine the prevalence of known disease-causing variants and rare variants of unknown clinical significance with a potential to adversely affect gene function in the leptin-melanocortin pathway in a large cohort of obese children, to assess the feasibility of genetic screening for monogenic obesity and to have the possibility to intervene early to more effectively prevent the development of obesity. Second, to analyse and predict the time-dependent trajectory of the early development of obesity and weight gain in participants with identified variants, not only in the homozygous state or in autosomal dominant genes, but also focusing on the contribution of rare heterozygous variants in autosomal recessive genes, which may occur more frequently in the population.

## Materials and Methods

### Study Population and Design

Children and adolescents born between 1998 and 2018, referred for evaluation for obesity to the central tertiary outpatient clinic were candidates for inclusion in the study with at least two visits in a clinic during the period 2013-2020 (n=1935). Data collected at the first examination were used for further analysis including participant’s family history of obesity, together with clinical anthropometric data, i.e. body weight, body mass index and age, assessed according to international reference values. Participants and/or their parents/guardians provided an estimate of the participant’s daily activity, defined as the number of days per week with the moderate activity of at least 45 minutes per day. In addition, self-reported data on eating habits was collected. The data included information on the number of menus planned by parents or school dietitians, which served as a proxy for diet regularity where meals and snacks were balanced, speed of eating as assessed by the self-perceived rate of eating on a scale of 1–3 (slow–fast), inclusion/exclusion of breakfast, frequency of drinking sugary drinks, and daily consumption of snacks and sweets.

**Figure 2:**
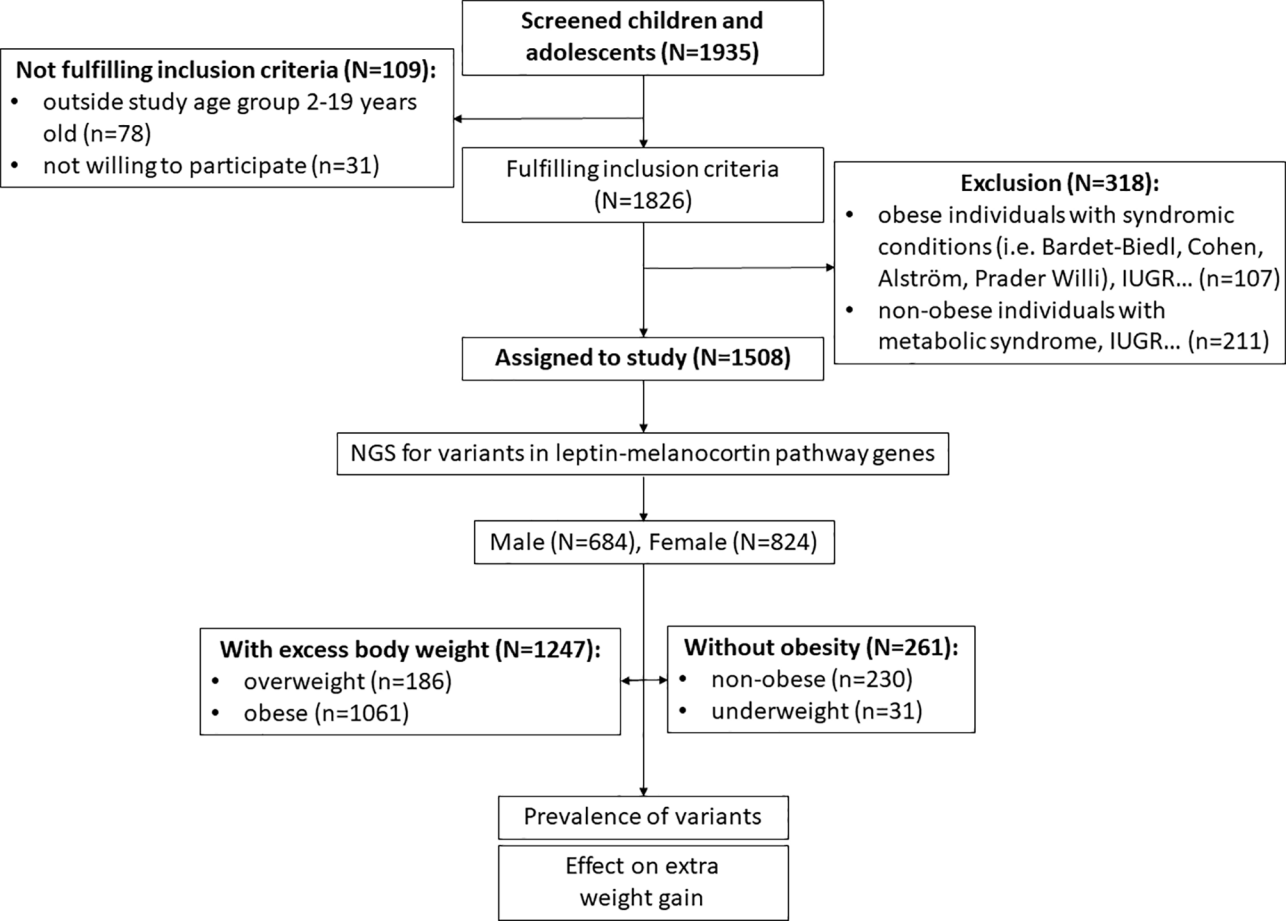
Recruitment process workflow.

In the first step of inclusion assessment, participants younger than 2 years and older than 19 years and those who did not sign a consent to participate were excluded (n=109). For the remaining participants (n=1826), BMI standard deviation scores (BMI SDS) accounted for participant sex and age were calculated from UK-WHO growth charts using “sds-function” as implemented in the R package “childsds” (17, 18). Based on calculated BMI SDS, groups of participants without obesity (underweight [BMI SDS<-2.0] and non-obese [-2.0≤BMI SDS<1.0]) and participants with excessive body weight (overweight [1.0≤BMI SDS<2.0] and obese [BMI SDS>2.0]) were created (1, 5, 19). According to the calculations, 472 subjects were identified as participants without obesity (BMI SDS<1) and 1354 subjects were identified as participants with excess body weight (BMI SDS>1). Next, we excluded 211 subjects from the cohort of participants without obesity who had clinical indications for metabolic syndrome components other than obesity (diabetes, hypercholesterolaemia, hypertension), intrauterine growth restriction (birth weight and/or height < 2 SDS) and hypopituitarism (defect in any of the hypothalamic-pituitary axis). No syndromic obesity cases (i.e. Bardet-Biedl syndrome, Cohen syndrome, Alström syndrome and Prader Willi syndrome) were identified in this group during clinical follow-up. On the other hand, among participants with excess body weight, we excluded subjects with syndromic obesity, those with hypopituitarism and those with intrauterine growth restriction (birth weight and/or height < 2 SDS) (n=107). Participants who might have additional clinical phenotypes unrelated to obesity, such as urinary tract impairment, respiratory failure and allergies, remained in the study. In the end, a total of 1247 children with excess body weight and 261 children without obesity aged 2-19 years were enrolled for downstream analysis (**Figure 2**).

The study was approved by the National Medical Ethics Committee of the Republic of Slovenia (25/10/09 and 29/2/13) and was conducted in accordance with the Declaration of Helsinki with all amendments. All parents or legal guardians gave written informed consent before inclusion in the study.

### Sequencing and Analysis

Genomic DNA was isolated from whole blood samples collected at 1^st^ visit at an outpatient clinic, using the FlexiGene isolation kit (Qiagen, Hilden, Germany). NGS libraries were prepared according to the manufacturer’s instructions, enriched with a modified gene panel and sequenced using the Illumina MiSeq sequencing platform (Illumina, San Diego, California). The gene panel included selected leptin and melanocortin pathway genes responsible for energy balance (*AGRP*, *LEP*, *LEPR*, *MC3R*, *MC4R*, *NPY*, *NPY1R*, *NPY5R*, *PCSK1*, *PCSK2*, *POMC*) (**Figure 1** and **Table 1**).

**Table 1:** Customized gene panel spanning genes involved in the hypothalamic leptin-melanocortin system, their clinical significance in regard to obesity and other metabolic syndromes, and inheritance patterns such as autosomal dominant (AD), autosomal recessive (AR) and mitochondrial inheritance (Mu). (10, 11, 20–28).

Gene	Name	Associated phenotypes	Inheritance	REF
*AGRP*	Agouti-related neuropeptide	Late-onset obesity	AD, AR, Mu	(23)
*LEP*	Leptin	Hereditary obesity, hyperphagia	AR	(11, 27)
*LEPR*	Leptin receptor	Obesity and pituitary dysfunction	AR	(22, 27)
*MC3R*	Melanocortin-3 receptor	Metabolic disorders	AD	(28)
*MC4R*	Melanocortin-4 receptor	Obesity, hyperphagia	AD	(10)
*NPY*	Neuropeptide Y	Metabolic disorders, early-onset of type 2 diabetes, obesity	NA	(24)
*NPY*1R	Neuropeptide Y receptor type 1	Metabolic disorders	NA	(25)
*NPY*5R	Neuropeptide Y receptor type 5	Dyslipidaemia, insulin resistance	NA	(25)
*PCSK1*	Pro-protein convertase type 1	Early-onset obesity, hyperphagia	AR	(26)
*PCSK2*	Pro-protein convertase type 2	Type 2 diabetes	NA	(20)
*POMC*	Proopiomelanocortin	Early-onset obesity, adrenal insufficiency	AR	(21)

A detailed bioinformatics pipeline with implemented statistical tools is described in the supplementary material. Briefly, data were filtered and aligned to the human reference hg19. Detected differences in genomic data (single nucleotide variants [SNVs]) and short insertions/deletions (indels) were functionally annotated and filtered for rare variants (minor allele frequency < 1%) and based on Combined Annotation Dependent Depletion (CADD) scores of “deleteriousness” (CADD > 20). Known homozygous, compound heterozygous or heterozygous mutations reported in the Human Gene Mutation Database (HGMD) (Institute of Medical Genetics, School of Medicine, University of Cardiff, Cardiff, Wales) and the ClinVar database (29) with a disease-causing association were classified as pathogenic, while the remainder were classified as variants of unknown clinical significance (VUS).

### Statistical Analysis

The required minimum sample size of 1067 participants was calculated with a 95% confidence level, a 3% margin of error, a population proportion of 0.5 and unlimited population size. Descriptive statistical methods were used to characterize the study population. Differences in BMI SDS scores between participants with versus without obesity and those with and without identified genetic variants were described using the Kruskal-Wallis test. Differences in the incidence of the identified variants in leptin-melanocortin pathway genes between groups with and without obesity, and the corresponding odds ratio (OR) distributions (95% confidence interval [CI] for OR) were calculated using Fisher’s exact test on 2x2 contingency tables. To mitigate the imbalance in the investigated groups R package ROSE: A Package for Binary Imbalanced Learning was utilized (30, 31). The minority class of participants without obesity was oversampled with replacement and the majority class of participants with excess body weight was under-sampled without replacement. In this way, different distribution of the two classes due to the randomness of the data generation was obtained (30). The procedure of resampling was iterated 9999-times. Statistical corrections for multiple comparisons were made using the Benjamini-Hochberg False Discovery Rate (FDR) correction for multiple testing. The calculated p-values for each gene were presented as histograms, defined by the median and interquartile range (IQR). In the case of statistically significant differences in the incidence of the variant in subjects with and without obesity, the majority of simulations were statistically significant and therefore a right-skewed distribution of the p-value histogram was obtained. Genes were associated with excess body weight if the median peak of the p-value distribution was below p<0.05, were partially associated if the IQR of the p-value distribution encompassed p=0.05, or were not associated with excess body weight if the overall IQR was higher than p=0.05. If the number of identified variants was equal to or less than 4, the calculation of the distribution of differences was not applicable. Next, effect size modelling of rare variants in the leptin-melanocortin signalling pathway on longitudinal weight gain was performed between subjects with an identified variant in the investigated gene and all subjects without an identified variant in this gene as well as in the other investigated genes. First, the mean BMI SDS of carriers of variants was calculated. If more than 4 variants were identified, 4 of them were randomly selected. Second, from all subjects in the study where no variants were identified, the mean BMI SDS among 4 random subjects was calculated. Finally, effect size on BMI SDS due to the identified variant was calculated by subtracting BMI SDS of non-carriers from BMI SDS of carriers of variants. The calculations were repeated 9999-times. Simulation of weight gain was performed by addition of the calculated effect sizes of BMI SDS to the data points from UKWHO growth percentile tables relative to body weight at the 50th percentile of height for statistically significant genes separately for females and males (1, 5, 19).

## Results

After the clinical examination in a tertiary paediatric outpatient clinic, 1508 children and adolescents born between 1998 and 2018 were eligible to participate in our study, and 427 were excluded due to age (younger than 2 years or older than 19 years) or other exclusion criteria such as intrauterine growth restriction, hypopituitarism (defect in any of the hypothalamic-pituitary axis), syndromic obesity cases, and metabolic syndrome components other than obesity (**Figure 2**). Of the 1247 participants with excess body weight, 1079 (84.6%) were born between 1998 and 2013. During these 15 years, the number of children and adolescents aged 2-19 years was 293,897 with an estimated prevalence of obesity of 6% (4.9% and 7.17%, for girls and boys, respectively) (32). Consequently, the estimated number of children and adolescents with obesity was approximately 17,633. The cohort recruited therefore represented 6.1% ± 3.8% of children and adolescents with obesity at the national level (Statistical Office of the Republic of Slovenia; https://www.stat.si/StatWeb).

### Genetic Characterisation

NGS was performed in all 1508 participants. 21 (1.4%) participants were found to have a heterozygous variant previously reported to cause disease (DCV). All were classified as obese, with a significantly higher BMI SDS compared to the other groups [BMI SDS index = 3.3 (0.9); median (IQR); (p ≤ 0.01)]. No DCVs were found in participants without obesity. Variants classified as VUS were found in additional 62 (4.1%) participants; 56 (3.7%) with excess body weight and 6 (0.4%) without obesity. Combined participants with and withouth obesity without VUS did not differ in BMI SDS from the corresponding groups of combined participants with and withouth obesity with VUS (**Table 2** and **Figure S1**). There was a statistically significant difference (p < 0.05) when comparing groups with and without obesity, and no statistical difference in age between groups with and without the identified variants (p = 0.257). Although the prevalence of obesity is higher in males, than in females as shown in Slovenia as well in other countries, there were no differences in the frequency of identified variants between female and male participants (p=0.304) (7, 32–34). The estimated incidence of genetic variants associated with excess body weight in our study of the Slovenian child population was 1/850 (1/2040 to 1/500) newborns for DCV alone and 1/150 (1/430 to 1/100) newborns for DCV and VUS combined.

**Table 2:** Identification of rare disease-causing variants (DCV) and rare variants with unknown clinical significance (VUS) in the leptin-melanocortin signalling pathway in participants with and without obesity.

		Total	Without obesity	With excess body weight
**Total**	Participants	1508 (100.0)	261 (17.3)	1247 (82.7)
	Female (%)	824 (54.6)	134 (8.9)	690 (45.8)
	Age [Median (IQR)]	12.5 (5.7)	9.6 (7.3)	12.8 (5.2)
	BMI SDS [Median (IQR)]	2.6 (1.3)	-0.1 (1.3)	2.8 (0.9)
**DCV**	Participants	21 (1.4)	/	21 (1.4)
	Female (%)	11 (0.7)	/	11 (0.7)
	Age [Median (IQR)]	14.0 (5.8)	/	14.0 (5.8)
	BMI SDS [Median (IQR)]	3.3 (0.9)	0 (0.0)	3.3 (0.9)
**VUS**	Participants	62 (4.1)	6 (0.4)	56 (3.7)
	Female (%)	31 (2.1)	3 (0.2)	28 (1.9)
	Age [Median (IQR)]	12.3 (5.7)	13.7 (7.8)	12.3 (5.5)
	BMI SDS [Median (IQR)]	2.7 (1.2)	0.7 (1.6)	2.7 (0.9)
**No variant**	Participants	1425 (94.5)	255 (16.9)	1170 (77.6)
	Female (%)	782 (51.9)	131 (8.7)	651 (43.2)
	Age [Median (IQR)]	12.5 (5.7)	9.6 (7.3)	12.8 (5.1)
	BMI SDS [Median (IQR)]	2.6 (1.4)	0.1 (1.2)	2.8 (0.9)

When following the family history of obesity, where the mother, father or both parents of the participants were living with excess body weight, no statistical difference was observed in our study population between participants with excess body weight and identified variant (DCV, VUS) and participants with excess body weight and no identified variants. Considering participants’ exercise habits, assessed as days of the week when participants were moderately active for at least 45 min per day, and dietary habits, assessed by self-assessment of the number of menus per day planned by parents or school dietitians, the pace of eating, the inclusion of breakfast, consumption of sugary drinks, snacks and sweets, no statistically significant differences were observed between the study groups (**Table 3**).

**Table 3:** Family history of participants with excess body weight and self-reported exercise and eating habits.

		Identified variant (DCV, VUS) in participants with excess body weight	No identified variants in participants with excess body weight	Total	p^1^
**Nr. of participants**		83	1170	1253	
Reported data (%)		60.2%	64.1%	64.2%	
**Parental obesity**					
Mother	without obesity	59.6%	54.0%	57.0%	0.556
	with excess body weight	40.4%	46.0%	43.0%	
Father	without obesity	48.2%	56.0%	51.9%	0.423
	with excess body weight	51.8%	44.0%	48.1%	
Both parents	without obesity	31.6%	36.0%	33.6%	0.455
	with excess body weight	22.8%	30.0%	40.2%	
Only one parent with excess body weight		45.6%	34.0%	26.2%	
**Exercise (days per week)^2^**					
	Mean (SD)	2.0 (1.1)	2.4 (1.2)	2.2 (1.1)	0.112
**Self-reported feeding habits^3^**					
	Very Poor	23.2%	19.6%	21.6%	0.706
	Poor	33.9%	26.1%	30.4%	
	Average	28.6%	34.8%	31.4%	
	Good	14.3%	19.6%	16.7%	

^1^ Chi-square test for independence.

^2^ Days per week with moderate activity for at least 45 minutes per day.

^3^ Self-reported feeding habits criteria:

Very Poor: rarely planned menus, no breakfast, drinks sugary drink, eats fast, snacks and sweets every day.

Poor: rarely planned to 2 planned menus, breakfast occasionally, drinks water and sugary drinks, eats fast, snacks and sweets occasionally.

Average: 3-5 planned menus, breakfast occasionally, drinks water and rarely sugary drinks, snacks and sweets rarely.

Good: 3-5 planned menus, breakfast included, eats slowly, drinks water, no snacks and sweets.

### Incidence of Obesity Associated With Loss of One Allele of a Functional Gene

**Figure 3:**
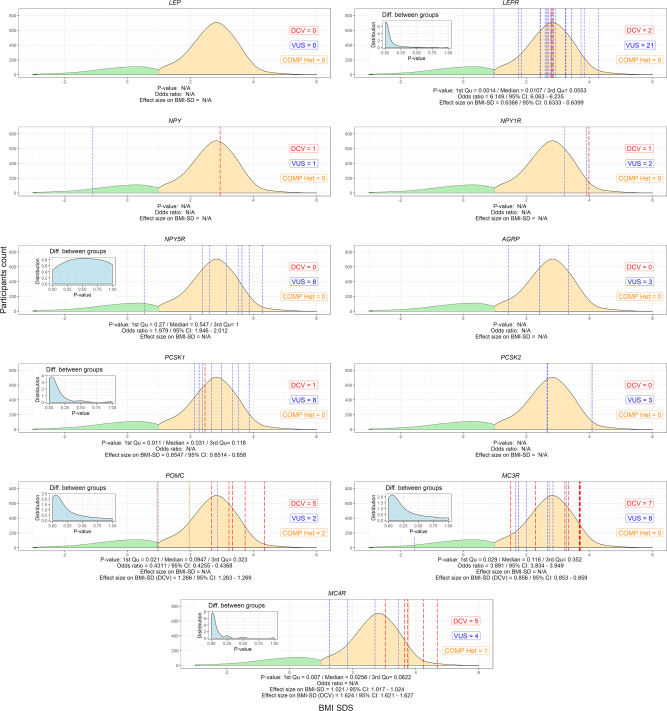
Variants in selected leptin-melanocortin pathway genes (*AGRP*, *LEP*, *LEPR*, *MC3R*, *MC4R*, *NPY*, *NPY1R*, *NPY5R*, *PCSK1*, *PCSK2*, and *POMC*). The x-axis shows the SDS body mass index and the y-axis shows the density of participants without (green) and with excess body weight (yellow). P-value distributions based on Fisher’s test for 2x2 contingency tables with 9999 permutations are presented in the upper left corner of the graphs and weighted by median and interquartile range, along with the calculated odds ratio and effect size on SDS BMI. Participants with identified disease-causing variants (DCV) (red), variants of unknown clinical significance (VUS) (blue) and compound heterozygotes (orange) at their respective BMI SDS are represented by vertical dashed lines. N/A, Not applicable.

No DCVs were found in the *LEP* gene. On the other hand, the prevalence of rare heterozygous variants found in the *LEPR* gene was 2% in obese participants. There was a significant difference in the number of carriers between the groups of children with and without obesity [p=0.0107 (0.0014-0.0553); median (IQR) of p-values distribution] for the *LEPR* gene, where 21 VUS and 2 DCVs were identified [BMI SDS=2.8 (0.6); median (IQR)]. 2 VUS and 2 compound VUS, identified in the *POMC* gene, did not cause a detectable difference in the number of carriers with and without obesity [BMI SDS=1.8 (1.6)], but 5 identified heterozygous DCVs in the *POMC* gene were identified in participants with excess body weight [BMI SDS=3.3 (0.5)] and none in participants without obesity. Significant differences were found in the number of carriers with and without obesity for the *PCSK1* gene [p=0.031 (0.011-0.118)], [BMI SDS=2.6 (0.7)]. Variants in the above autosomal recessive genes were present in 2.9% of the study population. Regarding autosomal dominant genes, a significant difference in the number of carriers with and without obesity was found for the *MC4R* gene [p=0.026 (0.007-0.0622]. Out of a total of 10 variants identified in the *MC4R* gene [BMI SDS=3.5 (1.0)], corresponding to 0.8% of the study population with a prevalence of 1/2000 children, 5 were heterozygous DCV [BMI SDS=3.7 (0.6)]. Similar to *MC4R*, *MC3R* was also identified as an important regulator of energy expenditure, with 7 DCVs [BMI SDS=3.3 (2.2)] showing early obesity, albeit with unclear difference between the number of carriers with and without obesity (**Figure 3** and **Table S1**).

### Effect of Variants in Genes Encoding the Leptin and Melanocortin Pathways on Weight Predisposition

**Figure 4:**
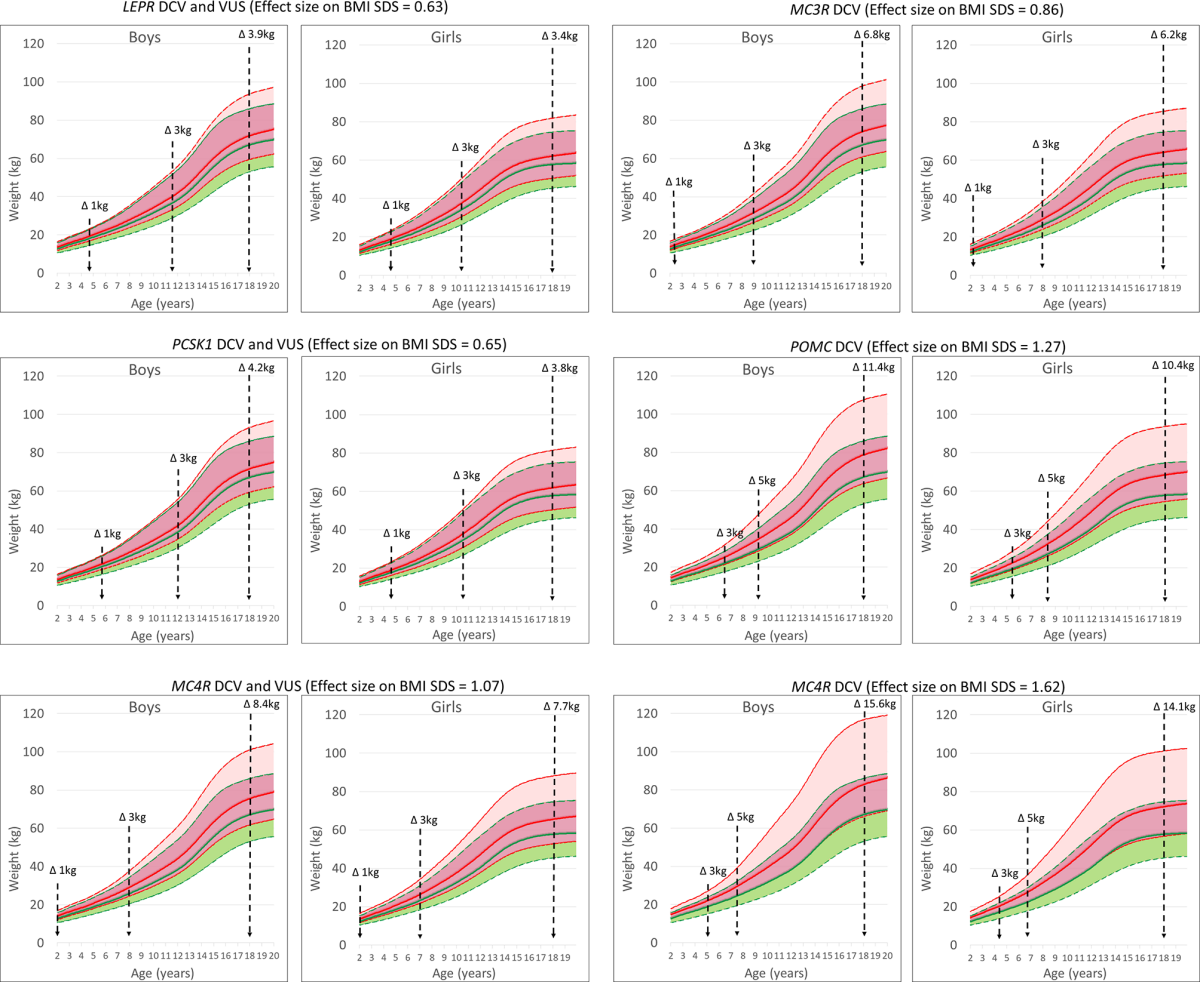
UKWHO growth percentile plots for boys and girls at the 50th percentile of height at age 2-19 years (green) and shifted BMI SDS effect size based on growth percentile plots for the *LEPR*, *POMC*, *PCSK1*, *MC3R* and *MC4R* gene variants (red). Vertical lines represent time points for weight change in participants.

Variants in obesity-related genes may alter the trajectory of an individual’s body weight towards an obesity phenotype. Carriers of VUS in the *LEPR* gene were at risk of a + 0.63 change in BMI SDS and approximately 3.4-3.9 kg of additional weight gain at 18 years (5.9-7.0 kg at the 95th percentile of body weight). A similar effect was found for variants in the *PCSK1* gene (3.8-4.2 kg). The effect size of a rare heterozygous *MC4R* variant that could affect function was +1.07 BMI SDS, or 7.7-8.4 kg additional bodyweight (14.2-15.9 kg at the 95th percentile of body weight). In addition, DCV in *MC4R* resulted in an effect size of +1.62 BMI SDS or 14.1-15.6 kg of additional body weight at age 18 years (26.1-30.3 kg at the 95th percentile of body weight). In addition, simulating the effect of DCV in the *POMC* gene and the *MC3R* gene resulted in approximately 10.4-11.4 kg (18.2-20.6 kg at the 95th percentile of body weight) and 6.2-6.8 kg (10.5-11.5 kg at the 95th percentile of body weight) of additional body weight gain, respectively, at age 18 years, compared to participants without the identified rare heterozygous variant. On average, the effect of the identified DCVs and VUS would result in 7.6 ± 4.1 kg (mean ± SD) and 8.4 ± 4.5 kg of additional body weight gain at age 18 years in girls and boys, respectively. In the study population at the 50th growth percentile, the calculated weight change in participants with an identified variant compared to all participants without an identified variant would exceed an additional 1 kg at approximately 5 years of age for *LEPR* and *PCSK1* and before 2 years of age for DCV in *MC3R*, *POMC* and *MC4R* (**Figure 4** and **Table S2**).

## Discussion

We showed that carriers of rare disease-causing heterozygous variants (1.4% of participants) and VUS (4.1% of participants) in the leptin-melanocortin pathway are predisposed to early obesity (with a median SDS BMI of 3.3 and 2.7, respectively) and prone to 7.6 kg (7.0-12.9 kg at the 95^th^ weight percentile) (girls) and 8.4 kg (8.2-14.4 kg) (boys) of additional weight gain compared to subjects without rare genetic variants in the leptin-melanocortin pathway. Although an obesogenic environment is a strong determinant of the development of obesity, children with a genetic predisposition and an altered pathway to the obesity phenotype should be identified at an early age with the first emergence of the clinical phenotype.

The compound heterozygous carriers of pathogenic genetic variants in the autosomal recessive genes *LEP*, *LEPR*, *POMC* and *PCSK1* are rare, obesity-associated variants in the autosomal dominant gene *MC4R* are the most common, with 0.8% of participants in our study carrying a variant in *MC4R* (13, 35, 36). We demonstrated a significant effect on additional weight gain, ranging from 8 kg for all identified variants to 15 kg for the DCV in *MC4R* at the end of adolescence. This effect on BMI SDS, calculated from LMS parameters by comparing the child’s measurements with the median size for a given age, allows for continuous prediction of weight gain throughout childhood and adolescence (17, 19). Furthermore, results of our modelled estimations are in accordance for loss of function in the *MC4R* gene measured by Wade et al. in the UK cohort where at age of 18 years, mean differences in body weight, body mass index and fat mass between carriers and non-carriers of LoF (DCV) mutations were 17.76kg (13, 35, 36). Our results showed that carriers of rare heterozygous variants in the leptin-melanocortin pathway are predisposed to the early onset of obesity. This effect of haploinsufficiency on body weight has been previously demonstrated for the *LEPR*, *MC3R*, *POMC* and *PCSK1* genes also in several other small population studies (12, 21, 26, 27, 35, 37–41). Our results additionally emphasize the importance of heterozygous potentially function-affecting genetic variants identification in autosomal recessive *LEPR*, *PCSK1* and *POMC*, as well as in autosomal dominant *MC3R* gene in children and adolescents, that drive the individual body mass trajectory towards the obese phenotype, either through involvement in food intake or in nutrient partitioning (7, 10, 12, 35, 37, 38, 41). Coinciding with the early onset of obesity, the predicted difference in body weight trajectory becomes evident from childhood growth charts before the age of five years. In addition, haploinsufficiency of autosomal recessive genes due to heterozygous DCV or rare VUS would result in an additional weight gain of approximately 4 kg in girls and boys at the end of adolescence for *LEPR* and *PCSK1* variants and of approximately 10 kg for carriers of heterozygous DCV in *POMC* gene.

In the field of obesity genetics, there is a strong population representation bias as most studies, like ours, have been carried out on populations of European origin (7). Nevertheless, genetic correlations between ancestries observed for BMI in populations of European, Asian, African, Latin American or other ancestry are broadly consistent with good transmissibility, although effect sizes and allele frequencies may vary (7). Consequently, early identification of individuals with DCV and rare VUS in leptin-melanocortin signalling pathway genes allows for a more accurate diagnosis of the type of obesity, which may prove useful regardless of individual’s ancestry and can be followed by tailored clinical intervention. To date, the administration of recombinant human leptin and the *MC4R* agonist setmelanotide are two genotype-informed therapies for rare monogenic obesity (7, 15, 42). For variants in the *POMC* gene, administration of setmelanotide reduced body weight by 25.6% approximately one year after the start of treatment (15, 43). This is in line with the modelled estimate of the projected weight gain in our study, where a +20% increase in body weight was predicted due to DCVs in the *POMC* gene. In addition, an established genetic diagnosis may improve the identification of those at the highest risk of becoming overweight in the future. Therefore, newborn genetic screening for DCV and rare VUS in the *LEPR*, *PCSK1*, *POMC*, *MC3R* and *MC4R* genes could be considered as a practical option. Another potential option is an early genetic screening as a part of clinical practice for those children that significantly deviate from the standard growth/weight curve, at the beginning of obesity phenotype development (4, 7–10, 14, 16, 22, 36, 44). Although the main aim of genetic screening is to identify individuals at risk of severe obesity, some shortcomings need to be addressed. Firstly, as previously reported, negative or positive genetic results could have undesirable psychological consequences due to the neglect of the effect of health-promoting behaviours, which could further result in maladaptive coping strategies such as eating to suppress negative emotions and, consequently, a positive feedback loop (2, 4). Secondly, genetic screening can be an integral part of potential genetic discrimination, so maintaining the confidentiality of genetic information found and establishing national instruments to develop stronger surveillance and accountability frameworks are crucial (45).

Nevertheless, our study with a representative sample of children with obesity (~6%) suggests that approximately 1/150 (VUS and DCV) to 1/850 (DCV) children with a genetic predisposition to obesity could benefit from early clinical intervention. While health-promoting behavioural interventions usually result in short-term weight loss in identified carriers of rare variants, as opposed to more sustained improvement in obese peers without these variants, additional and/or specific (if available) clinical interventions could be provided (4, 9, 10, 14–16). For the remaining 94% of the obese population in whom no genetic factors in the leptin-melanocortin signalling pathway have been identified, a potentially more ambitious clinical target could be set. In addition, individuals with extremely severe obesity in young adulthood with negative results from genetic analysis of the leptin-melanocortin signalling pathway may benefit from a large-scale genetic investigation involving whole exome or whole genome sequencing (8, 9).

Our study has some limitations. The cohort of subjects without obesity represents only 1/5 of all enrolled participants. This skew in the sample population towards children with excess body weight has several implications when attempting to assess the data presented. Therefore, repeated random sampling of participants from the obese and non-obese groups was adopted to mitigate the difference in group sizes and, in addition, to generate statistical significance values presented as histograms, along with estimates of specific effect sizes and their upper and lower confidence intervals. We anticipate that the estimated effect size of the BMI SDS of candidate variants affecting function in the genes analysed, obtained from the iterative analysis of our cohort, can be used in the UKWHO percentile growth tables to simulate the potential for weight gain and to show the weight gain trajectory of variant carriers. As such, the calculated weight trajectories are only an indirect representation of the potential shift in longitudinal weight gain. Nevertheless, recent reports support the results obtained, as discussed above. It is challenging to accurately measure people’s usual physical activity, since studies that measure physical activity more objectively (using accelerometers) suggest that people tend to overestimate their levels of activity. The same goes for the examination of self-reported feeding habits. Nevertheless, to address this issue, the table with percentages of parental obesity, the number of week-days when the subjects report activity for at least 45 minutes a day, and self-reported feeding habits were compiled from participant’s documentation, acquired during physical examinations at a tertiary outpatient clinic. Although other known obesity-related genes should be considered in genetic screening for obesity, the gene panel used provides a rationale for early detection of genetic determinants of early obesity and extreme hyperphagia, as recommended by the Endocrine Society (46).

## Conclusions

In addition to rare variants in the autosomal dominant *MC4R* gene associated with childhood obesity, heterozygous variants in the *LEPR*, *PCSK1*, *MC3R* and *POMC* genes were associated with significant weight gain in childhood. Individuals with haploinsufficiency in these genes could be identified in early childhood and their adverse weight trajectory reversed by innovative clinical approaches. Therefore, widespread genetic screening for variants of the *LEPR*, *PCSK1*, *POMC*, *MC4R* and *MC3R* genes could bring long-term clinical benefits.

## Data Availability Statement

The datasets presented in this study can be found in online repositories. The names of the repository/repositories and accession number(s) can be found below: https://www.ncbi.nlm.nih.gov/bioproject/PRJNA805092/.

## Ethics Statement

The studies involving human participants were reviewed and approved by Slovenian National Medical Ethics Committee (25/10/09 and 29/2/13). Written informed consent to participate in this study was provided by the participants’ legal guardian/next of kin.

## Author Contributions

Conceptualization, JK, PK, MD, and TB. Methodology, RŠ and JK. Software, RŠ. Validation, RŠ, BJB, TT, and JK. Formal analysis, RŠ and JK. Investigation, RŠ and VK. Data curation, RŠ, VK, MM, and BJB. Writing-original draft preparation, RŠ. Writing-review and editing, JK, PK, BJB, TT, VK, MM, MD, and TB. Visualization, RŠ. Supervision, JK. Funding acquisition, JK and TB. All authors have read and agreed to the published version of the manuscript.

## Funding

RŠ, JK, and TB report grants J3-9282, Z3-7412 and P3-0343 from the Slovenian Public Agency for Research during the study period.

## Conflict of Interest

The authors declare that the research was conducted in the absence of any commercial or financial relationships that could be construed as a potential conflict of interest.

## Publisher’s Note

All claims expressed in this article are solely those of the authors and do not necessarily represent those of their affiliated organizations, or those of the publisher, the editors and the reviewers. Any product that may be evaluated in this article, or claim that may be made by its manufacturer, is not guaranteed or endorsed by the publisher.
