# Supplementary material for: Heterozygous Genetic Variants in Autosomal Recessive Genes of the Leptin-Melanocortin Signalling Pathway Are Associated With the Development of Childhood Obesity
Source: Front Endocrinol (Lausanne). 2022 Apr 29;13:832911. doi: 10.3389/fendo.2022.832911 (PMC9105721; doi:10.3389/fendo.2022.832911)
Supplement: Supplementary file 1 [file DataSheet_1.pdf]

SUPPLEMENTARY MATERIAL

Heterozygous genetic variants in autosomal recessive genes of the leptin-melanocortin signalling pathway are associated with the development of childhood obesity

Robert Šket [1], Primož Kotnik [2, 3], Barbara Jenko Bizjan [1], Valentina Kocen [1], Matej Mlinarič [2], Tine Tesovnik [1], Maruša Debeljak [1,3], Tadej Battelino [2,3], Jernej Kovač [1,3,*]

[1] Clinical Institute of Special Laboratory Diagnostics, UMC - University Children's Hospital, University Medical Centre Ljubljana, Ljubljana, Slovenia
[2] Department of Pediatric Endocrinology, Diabetes and Metabolic Diseases, UMC - University Children's Hospital, University Medical Centre Ljubljana, Ljubljana, Slovenia
[3] Faculty of Medicine, University of Ljubljana, Ljubljana, Slovenia
*****Correspondence: jernej.kovac@kclj.si; Tel.: 0038615224023

Table S1: List of identified variants.

Count	Chr	Start	End	Ref	Alt	Gene	Exonic. Func.	avsnp 150	ACMG Classification	CADD Phred	AAChange.refgene	Participant's BMI SDS
1	chr20	17339041	17339041	G	A	PCSK2	nonsynonymous SNV	.	VUS (PM2, BP4)	21.8	PCSK2:NM_001201529:exon2:c.247G>A:p.D83N	2.65
1	chr20	17434591	17434591	G	A	PCSK2	nonsynonymous SNV	rs777932064	VUS (PM2)	24.8	PCSK2:NM_001201529:exon8:c.985G>A:p.E329K	2.67
1	chr20	17462477	17462477	G	T	PCSK2	nonsynonymous SNV	.	VUS (PM2)	31	PCSK2:NM_001201529:exon11:c.1574G>T:p.W525L	4.08
4	chr2	25384048	25384048	G	C	POMC	nonsynonymous SNV	rs28932472	Likely Pathogenic (PP3, BP6) *	28.1	POMC:NM_000939:exon3:c.706C>G:p.R236G	3.22; 3.73; 3.33; 4.35
2	chr2	25384138	25384138	C	A	POMC	stopgain	rs202127120	VUS (PVS1, BS2) DM?	40	POMC:NM_000939:exon3:c.616G>T:p.E206X	1.96; 0.94
2	chr2	25384149	25384149	-	GGGCCC	POMC	nonframeshift insertion	rs762710034	VUS (PM4, BS2)	22	POMC:NM_000939:exon3:c.604_605insGGGCCC:p.Q202delinsRAQ	1.96; 0.94
2	chr2	25384456	25384456	-	GCCGCTGCTGCCGCTGCT	POMC	nonframeshift insertion	rs10654394	VUS (PM4, BS1)	24.5	POMC:NM_000939:exon3:c.297_298insAGCAGCGGCAGCAGCGGC:p.A100delinsSSGSSGA	0.97; 2.86
1	chr2	25384458	25384458	C	-	POMC	frameshift deletion	.	Likely Pathogenic (PVS1, PM2) *	23.1	POMC:NM_000939:exon3:c.296delG:p.G99fs	2.67
1	chr20	54824272	54824272	T	C	MC3R	nonsynonymous SNV	.	VUS (PM2)	27.8	MC3R:NM_019888:exon1:c.373T>C:p.C125R	1.65
1	chr20	54824351	54824351	G	A	MC3R	nonsynonymous SNV	rs368067760	VUS (PM2)	31	MC3R:NM_019888:exon1:c.452G>A:p.R151H	-1.56
3	chr20	54824402	54824402	T	A	MC3R	nonsynonymous SNV	rs753068050	VUS (PM2, PP3)	27.8	MC3R:NM_019888:exon1:c.503T>A:p.I168N	2.84; 1.99. 2.67
1	chr20	54824557	54824557	C	A	MC3R	nonsynonymous SNV	rs61735259	VUS (BS2)	22.7	MC3R:NM_019888:exon1:c.658C>A:p.R220S	1.75
1	chr20	54824593	54824593	C	T	MC3R	nonsynonymous SNV	.	VUS (PM2, BP4)	22.4	MC3R:NM_019888:exon1:c.694C>T:p.P232S	3.27
1	chr20	54824629	54824629	A	G	MC3R	nonsynonymous SNV	.	VUS (PM2, BP4)	23.8	MC3R:NM_019888:exon1:c.730A>G:p.I244V	2.72
1	chr20	54824664	54824664	G	A	MC3R	stopgain	rs756089138	Likely Pathogenic (PM2)	41	MC3R:NM_019888:exon1:c.765G>A:p.W255X	3.71
6	chr20	54824791	54824791	AT	TC	MC3R	nonsynonymous SNV	.	Likely Pathogenic (PM2, PP2) *	26.6	MC3R:NM_019888:exon1:c.892_893delinsTC:p.I298S	3.22; 3.34; 3.69; 3.67; 1.5; 2.29

**1**	chr18	58038760	58038760	G	A	MC4R	nonsynonymous SNV	rs201813179	Likely Pathogenic (PM2, PM1, PP5) *	24.4	MC4R:NM_005912:exon1:c.823C>T:p.P275S	4.68
**1**	chr18	58038780	58038780	T	C	MC4R	nonsynonymous SNV	.	VUS (PM2, PM1, PP2)	26.8	MC4R:NM_005912:exon1:c.803A>G:p.Y268C	1.83
1	chr18	58038949	58038952	AGAG	-	MC4R	frameshift deletion	rs13447338	Likely Pathogenic (PVS1, PM2, PP2) *	33	MC4R:NM_005912:exon1:c.631_634del:p.L211fs	3.03
1	chr18	58039041	58039041	C	T	MC4R	nonsynonymous SNV	rs13447333	Likely Pathogenic (PM2, PM1, PP2, PP5) *	24.5	MC4R:NM_005912:exon1:c.542G>A:p.G181D	3.73
1	chr18	58039062	58039062	C	T	MC4R	stopgain	rs878909905	Likely Pathogenic (PVS1, PM2)	40	MC4R:NM_005912:exon1:c.521G>A:p.W174X	4.25
1	chr18	58039089	58039089	C	T	MC4R	nonsynonymous SNV	rs747681609	Pathogenic (PP5, PM2, PM1, PM5, PP2)	31	MC4R:NM_005912:exon1:c.494G>A:p.R165Q	3.65
**1**	chr18	58039167	58039167	C	A	MC4R	nonsynonymous SNV	.	VUS (PM2, PM1, PP2)	25.5	MC4R:NM_005912:exon1:c.416G>T:p.S139I	3.78
4	chr18	58039564	58039564	G	A	MC4R	nonsynonymous SNV	rs753796542	VUS (PM2, PP2, BP4)	20.9	MC4R:NM_005912:exon1:c.19C>T:p.R7C	3.78; 1.27; 2.71; 3.45
1	chr1	66036288	66036288	C	G	LEPR	stopgain	.	Likely Pathogenic (PVS1, PM2)	34	LEPR:NM_001198687:exon3:c.173C>G:p.S58X	2.82
1	chr1	66064439	66064439	C	A	LEPR	nonsynonymous SNV	rs754897895	VUS (PM2, BP4)	22.2	LEPR:NM_001198687:exon7:c.946C>A:p.P316T	2.59
1	chr1	66067141	66067141	A	G	LEPR	nonsynonymous SNV	rs183083824	VUS (PM2)	25.6	LEPR:NM_001198687:exon8:c.1061A>G:p.Y354C	2.93
1	chr1	66067305	66067305	G	T	LEPR	nonsynonymous SNV	.	VUS (PM2, PM5)	25.3	LEPR:NM_001198687:exon8:c.1225G>T:p.A409S	1.74
1	chr1	66067544	66067544	C	G	LEPR	stopgain	.	Likely Pathogenic (PVS1, PM2)	37	LEPR:NM_001198687:exon9:c.1304C>G:p.S435X	2.79
2	chr1	66070891	66070891	C	T	LEPR	nonsynonymous SNV	rs144310304	VUS (PM2, BP4)	20.8	LEPR:NM_001198687:exon10:c.1574C>T:p.P525L	3.73; 0.97
14	chr1	66083694	66083694	G	A	LEPR	nonsynonymous SNV	rs150936702	VUS (PM2)	27.2	LEPR:NM_001198687:exon15:c.2260G>A:p.V754M	2.7; 1.83; 2.75; 2.85; 3.83; 3.25; 4.28; 2.93 ; 3.42; 2.66; 3.42 ; 2.43; 3.24; 2.64
1	chr1	66102555	66102555	A	G	LEPR	nonsynonymous SNV	.	VUS (PM2, BP4)	20.3	LEPR:NM_002303:exon20:c.3355A>G:p.S1119G	2.45
1	chr1	66102613	66102613	T	C	LEPR	nonsynonymous SNV	.	VUS (PM2)	21.9	LEPR:NM_002303:exon20:c.3413T>C:p.F1138S	3.24
3	chr16	67516694	67516694	G	A	AGRP	nonsynonymous SNV	rs987185746	VUS (PM2)	31	AGRP:NM_001138:exon4:c.244C>T:p.R82C	2.41; 1.43; 3.34
1	chr5	95728728	95728728	C	-	PCSK1	frameshift deletion	.	VUS (PM2, PVS1)	32	PCSK1:NM_000439:exon14:c.2239delG:p.D747fs	2.46
1	chr5	95730651	95730651	T	C	PCSK1	nonsynonymous SNV	.	VUS (PM2)	22.7	PCSK1:NM_000439:exon13:c.1801A>G:p.M601V	2.12
1	chr5	95734645	95734645	A	G	PCSK1	nonsynonymous SNV	.	VUS (PM2)	24.9	PCSK1:NM_000439:exon11:c.1526T>C:p.F509S	3.53
1	chr5	95746489	95746489	C	T	PCSK1	nonsynonymous SNV		VUS (PM2)	31	PCSK1:NM_000439:exon8:c.1084G>A:p.D362N	3.86
1	chr5	95746573	95746573	C	T	PCSK1	nonsynonymous SNV	.	VUS (PM2)	24.5	PCSK1:NM_000439:exon8:c.1000G>A:p.A334T	3.33
2	chr5	95751745	95751745	T	A	PCSK1	nonsynonymous SNV	.	VUS (PM2, PP3)	28	PCSK1:NM_000439:exon6:c.701A>T:p.K234I	2.67; 2.90
2	chr5	95761624	95761624	G	A	PCSK1	nonsynonymous SNV	rs776019151	VUS (PM2)	23.5	PCSK1:NM_000439:exon3:c.296C>T:p.A99V	2.98; 2.37

1	chr7	24331300	24331300	-		NPY	stopgain	.	Likely Pathogenic (PM2)	35	NPY:NM_000905:exon4:c.288_289insTGGTGATGGGAAATGAGACT:p.M96_W97delinsMWX	2.94
1	chr7	24329140	24329140	G	A	NPY	nonsynonymous SNV	.	VUS (PM2)	26.9	NPY:NM_000905:exon3:c.211G>A:p.E71K	-1.10
1	chr4	1.64E+08	1.64E+08	G	A	NPY1R	stopgain	.	Likely Pathogenic (PM2)	38	NPY1R:NM_000909:exon2:c.358C>T:p.Q120X	3.98
2	chr4	1.64E+08	1.64E+08	A	G	NPY1R	nonsynonymous SNV	rs147899436	VUS (PM2)	25	NPY1R:NM_000909:exon2:c.128T>C:p.L43S	3.91; 3.20
1	chr4	1.64E+08	1.64E+08	G	A	NPY5R	nonsynonymous SNV	.	VUS (PM2)	25.8	NPY5R:NM_001317091:exon4:c.467G>A:p.G156D	2.38
7	chr4	1.64E+08	1.64E+08	C	T	NPY5R	nonsynonymous SNV	.	VUS (PM2, BP4)	23.6	NPY5R:NM_001317091:exon4:c.1060C>T:p.R354C	3.86; 4.28; 3.51; 0.53; 3.14; 3.63; 2.61

* ACMG criteria for pathogenicity was curated based on available data from Human Gene Mutation Database (http://www.hgmd.cf.ac.uk) and ClinVar (http://www.ncbi.nlm.nih.gov/clinvar) (Richards et al., 2015).

Table S2: Effect of variants in genes encoding the leptin-melanocortin pathway on weight predisposition in kg at 50th percentile of height and 50th percentile of weight for boys and girls from 2 to 20 years old based on UKWHO growth percentile tables (de Onis et al., 2012; Flegal and Cole, 2013; World Health Organization, 2019).

**Gene**	***LEPR* DCV and VUS**		***PCSK1* DCV and VUS**		***MC3R* DCV**		***POMC* DCV**		***MC4R* DCV and VUS**		***MC4R* DCV**	
	**Difference in weight (kg)**											
**Age (years)**	**Boys**	**Girls**	**Boys**	**Girls**	**Boys**	**Girls**	**Boys**	**Girls**	**Boys**	**Girls**	**Boys**	**Girls**
2.0	0.6	0.6	0.7	0.7	0.9	1.0	1.4	1.5	1.1	1.1	1.8	1.9
2.1	0.6	0.6	0.7	0.7	1.0	1.0	1.5	1.5	1.1	1.2	1.9	1.9
2.2	0.6	0.6	0.7	0.7	1.0	1.0	1.5	1.5	1.2	1.2	1.9	1.9
2.3	0.6	0.6	0.7	0.7	1.0	1.0	1.5	1.5	1.2	1.2	1.9	2.0
2.3	0.6	0.6	0.7	0.7	1.0	1.0	1.5	1.6	1.2	1.2	1.9	2.0
2.4	0.6	0.7	0.7	0.7	1.0	1.0	1.5	1.6	1.2	1.2	2.0	2.0
2.5	0.6	0.7	0.7	0.7	1.0	1.0	1.6	1.6	1.2	1.2	2.0	2.1
2.6	0.6	0.7	0.7	0.7	1.0	1.1	1.6	1.6	1.2	1.3	2.0	2.1
2.7	0.7	0.7	0.7	0.7	1.0	1.1	1.6	1.6	1.2	1.3	2.0	2.1
2.8	0.7	0.7	0.7	0.7	1.0	1.1	1.6	1.7	1.2	1.3	2.1	2.2
2.8	0.7	0.7	0.7	0.8	1.1	1.1	1.6	1.7	1.3	1.3	2.1	2.2
2.9	0.7	0.7	0.7	0.8	1.1	1.1	1.6	1.7	1.3	1.3	2.1	2.2
3.0	0.7	0.7	0.7	0.8	1.1	1.1	1.7	1.7	1.3	1.3	2.1	2.2
3.1	0.7	0.7	0.7	0.8	1.1	1.1	1.7	1.8	1.3	1.4	2.2	2.3
3.2	0.7	0.7	0.7	0.8	1.1	1.1	1.7	1.8	1.3	1.4	2.2	2.3
3.3	0.7	0.7	0.7	0.8	1.1	1.2	1.7	1.8	1.3	1.4	2.2	2.3
3.3	0.7	0.7	0.8	0.8	1.1	1.2	1.7	1.8	1.3	1.4	2.2	2.4
3.4	0.7	0.7	0.8	0.8	1.1	1.2	1.7	1.8	1.3	1.4	2.2	2.4
3.5	0.7	0.7	0.8	0.8	1.1	1.2	1.7	1.9	1.3	1.4	2.3	2.4
3.6	0.7	0.7	0.8	0.8	1.1	1.2	1.8	1.9	1.4	1.4	2.3	2.5
3.7	0.7	0.7	0.8	0.8	1.1	1.2	1.8	1.9	1.4	1.5	2.3	2.5
3.8	0.7	0.7	0.8	0.8	1.1	1.2	1.8	1.9	1.4	1.5	2.3	2.5
3.8	0.7	0.7	0.8	0.8	1.2	1.2	1.8	2.0	1.4	1.5	2.4	2.6
3.9	0.7	0.8	0.8	0.8	1.2	1.2	1.8	2.0	1.4	1.5	2.4	2.6
4.0	0.7	0.8	0.8	0.8	1.2	1.3	1.8	2.0	1.4	1.5	2.4	2.6
4.1	0.7	0.8	0.8	0.9	1.2	1.3	1.9	2.0	1.4	1.6	2.4	2.7
4.2	0.7	0.8	0.8	0.9	1.2	1.3	1.9	2.1	1.5	1.6	2.5	2.7

4.3	0.8	0.8	0.8	0.9	1.2	1.3	1.9	2.1	1.5	1.6	2.5	2.8
4.3	0.8	0.8	0.8	0.9	1.2	1.4	1.9	2.2	1.5	1.7	2.5	2.8
4.4	0.8	0.8	0.9	0.9	1.3	1.4	2.0	2.2	1.5	1.7	2.6	2.9
4.5	0.8	0.9	0.9	1.0	1.3	1.4	2.0	2.3	1.5	1.7	2.6	3.0
4.6	0.8	0.9	0.9	1.0	1.3	1.5	2.0	2.3	1.6	1.8	2.6	3.0
4.7	0.8	0.9	0.9	1.0	1.3	1.5	2.1	2.4	1.6	1.8	2.7	3.1
4.8	0.8	1.0	0.9	1.0	1.3	1.5	2.1	2.4	1.6	1.9	2.7	3.2
4.8	0.9	1.0	0.9	1.1	1.4	1.6	2.1	2.5	1.6	1.9	2.8	3.2
4.9	0.9	1.0	1.0	1.1	1.4	1.6	2.2	2.5	1.7	2.0	2.8	3.3
5.0	0.9	1.0	1.0	1.1	1.4	1.7	2.2	2.6	1.7	2.0	2.9	3.4
5.1	0.9	1.1	1.0	1.2	1.4	1.7	2.2	2.7	1.7	2.0	2.9	3.5
5.2	0.9	1.1	1.0	1.2	1.5	1.7	2.3	2.7	1.8	2.1	3.0	3.5
5.3	0.9	1.1	1.0	1.2	1.5	1.8	2.3	2.8	1.8	2.1	3.0	3.6
5.3	1.0	1.2	1.0	1.3	1.5	1.8	2.4	2.8	1.8	2.2	3.1	3.7
5.4	1.0	1.2	1.1	1.3	1.5	1.9	2.4	2.9	1.8	2.2	3.1	3.7
5.5	1.0	1.2	1.1	1.3	1.6	1.9	2.4	2.9	1.9	2.3	3.2	3.8
5.6	1.0	1.2	1.1	1.3	1.6	1.9	2.5	3.0	1.9	2.3	3.2	3.9
5.7	1.0	1.2	1.1	1.4	1.6	2.0	2.5	3.0	1.9	2.3	3.3	4.0
5.8	1.0	1.3	1.1	1.4	1.6	2.0	2.5	3.1	2.0	2.4	3.3	4.0
5.8	1.0	1.3	1.1	1.4	1.7	2.0	2.6	3.1	2.0	2.4	3.4	4.1
5.9	1.1	1.3	1.2	1.4	1.7	2.1	2.6	3.2	2.0	2.5	3.4	4.2
6.0	1.1	1.3	1.2	1.4	1.7	2.1	2.7	3.3	2.1	2.5	3.5	4.2
6.1	1.1	1.3	1.2	1.5	1.7	2.1	2.7	3.3	2.1	2.5	3.6	4.3
6.2	1.1	1.4	1.2	1.5	1.8	2.2	2.8	3.4	2.1	2.6	3.6	4.4
6.3	1.1	1.4	1.2	1.5	1.8	2.2	2.8	3.4	2.2	2.6	3.7	4.5
6.3	1.2	1.4	1.3	1.5	1.8	2.2	2.9	3.5	2.2	2.7	3.8	4.5
6.4	1.2	1.4	1.3	1.6	1.9	2.3	2.9	3.5	2.2	2.7	3.8	4.6
6.5	1.2	1.5	1.3	1.6	1.9	2.3	3.0	3.6	2.3	2.8	3.9	4.7
6.6	1.2	1.5	1.3	1.6	1.9	2.3	3.0	3.7	2.3	2.8	4.0	4.8
6.7	1.2	1.5	1.3	1.6	2.0	2.4	3.1	3.7	2.4	2.8	4.0	4.9
6.8	1.2	1.5	1.4	1.6	2.0	2.4	3.1	3.8	2.4	2.9	4.1	4.9
6.8	1.3	1.5	1.4	1.7	2.0	2.4	3.2	3.8	2.4	2.9	4.2	5.0
6.9	1.3	1.5	1.4	1.7	2.1	2.5	3.2	3.9	2.5	3.0	4.3	5.1
7.0	1.3	1.6	1.4	1.7	2.1	2.5	3.3	3.9	2.5	3.0	4.3	5.2
7.1	1.3	1.6	1.5	1.7	2.1	2.5	3.3	4.0	2.6	3.0	4.4	5.3
7.2	1.4	1.6	1.5	1.7	2.2	2.6	3.4	4.1	2.6	3.1	4.5	5.3
7.3	1.4	1.6	1.5	1.8	2.2	2.6	3.5	4.1	2.6	3.1	4.6	5.4
7.3	1.4	1.6	1.5	1.8	2.2	2.6	3.5	4.2	2.7	3.2	4.7	5.5
7.4	1.4	1.6	1.6	1.8	2.3	2.7	3.6	4.2	2.7	3.2	4.7	5.6
7.5	1.4	1.7	1.6	1.8	2.3	2.7	3.7	4.3	2.8	3.3	4.8	5.7
7.6	1.5	1.7	1.6	1.9	2.4	2.8	3.7	4.4	2.8	3.3	4.9	5.8
7.7	1.5	1.7	1.6	1.9	2.4	2.8	3.8	4.4	2.9	3.4	5.0	5.9
7.8	1.5	1.7	1.7	1.9	2.4	2.8	3.9	4.5	2.9	3.4	5.1	6.0
7.8	1.6	1.8	1.7	1.9	2.5	2.9	3.9	4.6	3.0	3.5	5.2	6.1
7.9	1.6	1.8	1.7	2.0	2.5	2.9	4.0	4.7	3.0	3.5	5.3	6.2
8.0	1.6	1.8	1.8	2.0	2.6	3.0	4.1	4.7	3.1	3.6	5.4	6.3
8.1	1.6	1.9	1.8	2.0	2.6	3.0	4.1	4.8	3.1	3.7	5.5	6.4
8.2	1.7	1.9	1.8	2.1	2.6	3.1	4.2	4.9	3.2	3.7	5.6	6.5
8.3	1.7	1.9	1.8	2.1	2.7	3.1	4.3	5.0	3.2	3.8	5.6	6.6
8.3	1.7	1.9	1.9	2.1	2.7	3.2	4.3	5.0	3.3	3.8	5.7	6.7
8.4	1.7	2.0	1.9	2.2	2.8	3.2	4.4	5.1	3.3	3.9	5.8	6.8
8.5	1.7	2.0	1.9	2.2	2.8	3.2	4.5	5.2	3.4	3.9	5.9	6.9
8.6	1.8	2.0	1.9	2.2	2.8	3.3	4.5	5.3	3.4	4.0	6.0	7.0

8.7	1.8	2.0	2.0	2.2	2.9	3.3	4.6	5.3	3.5	4.0	6.1	7.1
8.8	1.8	2.1	2.0	2.3	2.9	3.4	4.6	5.4	3.5	4.1	6.2	7.2
8.8	1.8	2.1	2.0	2.3	2.9	3.4	4.7	5.5	3.6	4.1	6.3	7.3
8.9	1.8	2.1	2.0	2.3	3.0	3.5	4.8	5.5	3.6	4.2	6.4	7.4
9.0	1.9	2.1	2.0	2.4	3.0	3.5	4.8	5.6	3.7	4.2	6.5	7.5
9.1	1.9	2.2	2.1	2.4	3.1	3.5	4.9	5.7	3.7	4.3	6.6	7.6
9.2	1.9	2.2	2.1	2.4	3.1	3.6	5.0	5.8	3.8	4.4	6.6	7.7
9.3	1.9	2.2	2.1	2.4	3.1	3.6	5.0	5.8	3.8	4.4	6.7	7.8
9.3	2.0	2.2	2.2	2.5	3.2	3.7	5.1	5.9	3.9	4.5	6.8	7.9
9.4	2.0	2.3	2.2	2.5	3.2	3.7	5.2	6.0	3.9	4.5	6.9	8.0
9.5	2.0	2.3	2.2	2.5	3.3	3.8	5.2	6.1	4.0	4.6	7.0	8.1
9.6	2.0	2.3	2.2	2.6	3.3	3.8	5.3	6.1	4.0	4.6	7.1	8.2
9.7	2.1	2.4	2.3	2.6	3.4	3.9	5.4	6.2	4.1	4.7	7.2	8.3
9.8	2.1	2.4	2.3	2.6	3.4	3.9	5.5	6.3	4.1	4.8	7.3	8.4
9.8	2.1	2.4	2.3	2.7	3.4	4.0	5.5	6.4	4.2	4.8	7.4	8.5
9.9	2.1	2.5	2.3	2.7	3.5	4.0	5.6	6.5	4.2	4.9	7.5	8.6
10.0	2.2	2.5	2.4	2.7	3.5	4.1	5.7	6.6	4.3	5.0	7.6	8.7
10.1	2.2	2.5	2.4	2.8	3.6	4.1	5.7	6.6	4.3	5.0	7.7	8.9
10.2	2.2	2.6	2.4	2.8	3.6	4.2	5.8	6.7	4.4	5.1	7.8	9.0
10.3	2.2	2.6	2.5	2.9	3.7	4.3	5.9	6.8	4.4	5.2	7.9	9.1
10.3	2.3	2.7	2.5	2.9	3.7	4.3	5.9	6.9	4.5	5.2	8.0	9.2
10.4	2.3	2.7	2.5	3.0	3.7	4.4	6.0	7.0	4.5	5.3	8.1	9.3
10.5	2.3	2.8	2.5	3.0	3.8	4.5	6.1	7.1	4.6	5.4	8.2	9.5
10.6	2.4	2.8	2.6	3.1	3.8	4.5	6.2	7.2	4.6	5.5	8.3	9.6
10.7	2.4	2.9	2.6	3.1	3.9	4.6	6.2	7.3	4.7	5.6	8.4	9.7
10.8	2.4	2.9	2.6	3.2	3.9	4.7	6.3	7.4	4.7	5.6	8.5	9.8
10.8	2.4	3.0	2.7	3.2	4.0	4.7	6.4	7.5	4.8	5.7	8.6	10.0
10.9	2.5	3.0	2.7	3.3	4.0	4.8	6.4	7.6	4.9	5.8	8.7	10.1
11.0	2.5	3.1	2.7	3.3	4.0	4.9	6.5	7.7	4.9	5.9	8.8	10.2
11.1	2.5	3.1	2.8	3.4	4.1	4.9	6.6	7.8	5.0	5.9	8.9	10.3
11.2	2.6	3.1	2.8	3.4	4.1	5.0	6.7	7.9	5.0	6.0	9.0	10.5
11.3	2.6	3.2	2.8	3.5	4.2	5.1	6.7	8.0	5.1	6.1	9.1	10.6
11.3	2.6	3.2	2.9	3.5	4.2	5.1	6.8	8.1	5.1	6.2	9.2	10.7
11.4	2.7	3.3	2.9	3.6	4.3	5.2	6.9	8.2	5.2	6.2	9.3	10.8
11.5	2.7	3.3	2.9	3.6	4.3	5.2	7.0	8.2	5.3	6.3	9.4	10.9
11.6	2.7	3.3	3.0	3.6	4.4	5.3	7.1	8.3	5.3	6.3	9.5	11.0
11.7	2.8	3.4	3.0	3.7	4.5	5.3	7.1	8.4	5.4	6.4	9.6	11.1
11.8	2.8	3.4	3.1	3.7	4.5	5.4	7.2	8.5	5.5	6.5	9.7	11.2
11.8	2.8	3.4	3.1	3.7	4.6	5.4	7.3	8.6	5.5	6.5	9.8	11.3
11.9	2.9	3.4	3.1	3.7	4.6	5.5	7.4	8.6	5.6	6.6	9.9	11.4
12.0	2.9	3.5	3.2	3.8	4.7	5.5	7.5	8.7	5.6	6.6	10.0	11.5
12.1	2.9	3.5	3.2	3.8	4.7	5.5	7.5	8.8	5.7	6.7	10.1	11.6
12.2	3.0	3.5	3.3	3.8	4.8	5.6	7.6	8.8	5.8	6.7	10.2	11.7
12.3	3.0	3.5	3.3	3.8	4.8	5.6	7.7	8.9	5.8	6.8	10.3	11.8
12.3	3.0	3.5	3.3	3.8	4.9	5.6	7.8	8.9	5.9	6.8	10.4	11.9
12.4	3.1	3.5	3.4	3.8	4.9	5.6	7.9	9.0	6.0	6.8	10.6	12.0
12.5	3.1	3.5	3.4	3.8	5.0	5.7	8.0	9.0	6.0	6.9	10.7	12.0
12.6	3.1	3.5	3.4	3.8	5.0	5.7	8.0	9.1	6.1	6.9	10.8	12.1
12.7	3.2	3.5	3.4	3.8	5.1	5.7	8.1	9.1	6.1	6.9	10.9	12.2
12.8	3.2	3.5	3.5	3.8	5.1	5.7	8.2	9.1	6.2	6.9	11.0	12.2
12.8	3.2	3.4	3.5	3.8	5.2	5.7	8.3	9.2	6.3	6.9	11.1	12.3
12.9	3.2	3.4	3.5	3.8	5.2	5.7	8.4	9.2	6.3	6.9	11.2	12.3
13.0	3.3	3.4	3.6	3.8	5.3	5.7	8.4	9.2	6.4	6.9	11.3	12.4

13.1	3.3	3.4	3.6	3.7	5.3	5.7	8.5	9.3	6.4	6.9	11.4	12.4
13.2	3.3	3.4	3.6	3.7	5.4	5.7	8.6	9.3	6.5	6.9	11.6	12.5
13.3	3.3	3.3	3.7	3.7	5.4	5.7	8.7	9.3	6.6	6.9	11.7	12.5
13.3	3.4	3.3	3.7	3.7	5.5	5.7	8.8	9.3	6.6	6.9	11.8	12.6
13.4	3.4	3.3	3.7	3.6	5.5	5.6	8.9	9.3	6.7	6.9	11.9	12.6
13.5	3.4	3.3	3.8	3.6	5.6	5.6	9.0	9.4	6.8	6.9	12.0	12.6
13.6	3.5	3.2	3.8	3.6	5.6	5.6	9.1	9.4	6.8	6.9	12.2	12.7
13.7	3.5	3.2	3.8	3.6	5.7	5.6	9.1	9.4	6.9	7.0	12.3	12.7
13.8	3.5	3.2	3.8	3.6	5.7	5.6	9.2	9.4	6.9	7.0	12.4	12.8
13.8	3.5	3.2	3.9	3.6	5.7	5.6	9.3	9.4	7.0	7.0	12.5	12.8
13.9	3.5	3.2	3.9	3.6	5.8	5.6	9.4	9.5	7.0	7.0	12.6	12.9
14.0	3.5	3.2	3.9	3.5	5.8	5.6	9.5	9.5	7.1	7.0	12.7	12.9
14.1	3.6	3.2	3.9	3.5	5.9	5.6	9.5	9.5	7.2	7.0	12.8	12.9
14.2	3.6	3.1	3.9	3.5	5.9	5.6	9.6	9.5	7.2	7.0	12.9	13.0
14.3	3.6	3.1	3.9	3.5	5.9	5.6	9.7	9.6	7.2	7.0	13.0	13.0
14.3	3.6	3.1	4.0	3.5	6.0	5.7	9.7	9.6	7.3	7.0	13.1	13.1
14.4	3.6	3.1	4.0	3.5	6.0	5.7	9.8	9.6	7.3	7.1	13.3	13.1
14.5	3.6	3.1	4.0	3.5	6.0	5.7	9.9	9.6	7.4	7.1	13.3	13.1
14.6	3.6	3.1	4.0	3.5	6.1	5.7	9.9	9.7	7.4	7.1	13.4	13.2
14.7	3.6	3.1	4.0	3.5	6.1	5.7	10.0	9.7	7.5	7.1	13.5	13.2
14.8	3.6	3.1	4.0	3.5	6.1	5.7	10.1	9.7	7.5	7.1	13.6	13.3
14.8	3.6	3.1	4.0	3.5	6.2	5.7	10.1	9.7	7.5	7.1	13.7	13.3
14.9	3.7	3.1	4.0	3.5	6.2	5.7	10.2	9.7	7.6	7.1	13.8	13.3
15.0	3.7	3.1	4.0	3.5	6.2	5.7	10.2	9.8	7.6	7.1	13.9	13.3
15.1	3.7	3.1	4.0	3.5	6.2	5.7	10.3	9.8	7.6	7.2	13.9	13.4
15.2	3.7	3.1	4.1	3.5	6.2	5.7	10.3	9.8	7.7	7.2	14.0	13.4
15.3	3.6	3.1	4.0	3.5	6.3	5.7	10.4	9.8	7.7	7.2	14.1	13.4
15.3	3.6	3.1	4.0	3.5	6.3	5.7	10.4	9.8	7.7	7.2	14.1	13.4
15.4	3.6	3.1	4.0	3.5	6.3	5.7	10.4	9.8	7.7	7.2	14.2	13.5
15.5	3.6	3.1	4.0	3.5	6.3	5.7	10.5	9.9	7.8	7.2	14.2	13.5
15.6	3.6	3.1	4.0	3.5	6.3	5.7	10.5	9.9	7.8	7.2	14.3	13.5
15.7	3.6	3.1	4.0	3.5	6.3	5.7	10.5	9.9	7.8	7.2	14.4	13.5
15.8	3.6	3.1	4.0	3.5	6.3	5.7	10.6	9.9	7.8	7.2	14.4	13.5
15.8	3.6	3.1	4.0	3.5	6.3	5.7	10.6	9.9	7.8	7.2	14.4	13.5
15.9	3.6	3.1	4.0	3.5	6.3	5.7	10.6	9.9	7.8	7.2	14.5	13.6
16.0	3.6	3.1	4.0	3.5	6.3	5.7	10.7	9.9	7.8	7.2	14.5	13.6
16.1	3.6	3.1	4.0	3.5	6.3	5.7	10.7	9.9	7.9	7.2	14.6	13.6
16.2	3.6	3.0	4.0	3.5	6.3	5.7	10.7	9.9	7.9	7.2	14.6	13.6
16.3	3.6	3.1	4.0	3.5	6.4	5.7	10.7	9.9	7.9	7.2	14.7	13.6
16.3	3.6	3.0	4.0	3.5	6.4	5.7	10.8	9.9	7.9	7.2	14.7	13.6
16.4	3.6	3.1	4.0	3.5	6.4	5.8	10.8	9.9	7.9	7.2	14.8	13.6
16.5	3.6	3.1	4.0	3.5	6.4	5.8	10.8	10.0	8.0	7.2	14.8	13.6
16.6	3.6	3.1	4.0	3.5	6.4	5.8	10.9	10.0	8.0	7.3	14.9	13.7
16.7	3.6	3.1	4.0	3.5	6.4	5.8	10.9	10.0	8.0	7.3	14.9	13.7
16.8	3.6	3.1	4.0	3.5	6.4	5.8	10.9	10.0	8.0	7.3	14.9	13.7
16.8	3.6	3.1	4.0	3.5	6.5	5.8	11.0	10.0	8.1	7.3	15.0	13.7
16.9	3.6	3.1	4.0	3.5	6.5	5.8	11.0	10.0	8.1	7.3	15.0	13.7
17.0	3.6	3.1	4.1	3.5	6.5	5.8	11.0	10.1	8.1	7.3	15.1	13.8
17.1	3.6	3.1	4.1	3.5	6.5	5.8	11.1	10.1	8.1	7.3	15.1	13.8
17.2	3.7	3.1	4.1	3.6	6.6	5.9	11.1	10.1	8.2	7.4	15.2	13.8
17.3	3.7	3.2	4.1	3.6	6.6	5.9	11.2	10.1	8.2	7.4	15.2	13.8
17.3	3.7	3.2	4.1	3.6	6.6	5.9	11.2	10.1	8.2	7.4	15.3	13.8
17.4	3.7	3.2	4.2	3.6	6.6	5.9	11.2	10.2	8.3	7.4	15.3	13.9

17.5	3.7	3.2	4.2	3.6	6.7	6.0	11.3	10.2	8.3	7.5	15.4	13.9
17.6	3.7	3.2	4.2	3.7	6.7	6.0	11.3	10.2	8.3	7.5	15.4	13.9
17.7	3.8	3.3	4.2	3.7	6.7	6.0	11.3	10.3	8.3	7.5	15.4	14.0
17.8	3.8	3.3	4.2	3.7	6.7	6.1	11.4	10.3	8.4	7.6	15.5	14.0
17.8	3.8	3.3	4.2	3.8	6.8	6.1	11.4	10.3	8.4	7.6	15.5	14.1
17.9	3.8	3.4	4.2	3.8	6.8	6.1	11.4	10.4	8.4	7.6	15.5	14.1
18.0	3.9	3.4	4.2	3.8	6.8	6.2	11.4	10.4	8.4	7.7	15.6	14.1
18.1	3.9	3.5	4.3	3.9	6.8	6.2	11.4	10.5	8.4	7.7	15.6	14.2
18.2	3.9	3.5	4.3	3.9	6.8	6.2	11.5	10.5	8.4	7.8	15.6	14.2
18.3	3.9	3.5	4.3	4.0	6.8	6.3	11.5	10.5	8.5	7.8	15.6	14.3
18.3	3.9	3.6	4.3	4.0	6.8	6.3	11.5	10.6	8.5	7.8	15.7	14.3
18.4	3.9	3.6	4.3	4.0	6.8	6.4	11.5	10.6	8.5	7.9	15.7	14.4
18.5	3.9	3.7	4.3	4.1	6.9	6.4	11.5	10.7	8.5	7.9	15.7	14.4
18.6	3.9	3.7	4.3	4.1	6.9	6.5	11.6	10.7	8.5	8.0	15.7	14.5
18.7	3.9	3.7	4.3	4.2	6.9	6.5	11.6	10.8	8.5	8.0	15.7	14.5
18.8	3.9	3.8	4.3	4.2	6.9	6.5	11.6	10.8	8.6	8.0	15.7	14.6
18.8	4.0	3.8	4.4	4.2	6.9	6.6	11.6	10.9	8.6	8.1	15.8	14.6
18.9	4.0	3.9	4.4	4.3	6.9	6.6	11.6	10.9	8.6	8.1	15.8	14.6
19.0	4.0	3.9	4.4	4.3	7.0	6.7	11.7	10.9	8.6	8.2	15.8	14.7
19.1	4.1	3.9	4.4	4.4	7.0	6.7	11.7	11.0	8.6	8.2	15.9	14.7
19.2	4.1	4.0	4.4	4.4	7.0	6.8	11.7	11.0	8.7	8.3	15.9	14.8
19.3	4.1	4.0	4.5	4.5	7.0	6.8	11.8	11.1	8.7	8.3	15.9	14.8
19.3	4.1	4.1	4.5	4.5	7.1	6.8	11.8	11.1	8.8	8.4	16.0	14.9
19.4	4.2	4.1	4.5	4.5	7.1	6.9	11.8	11.2	8.8	8.4	16.0	14.9
19.5	4.3	4.2	4.6	4.6	7.2	6.9	11.9	11.2	8.8	8.4	16.0	15.0
19.6	4.3	4.2	4.6	4.6	7.2	7.0	11.9	11.3	8.9	8.5	16.1	15.0
19.7	4.3	4.2	4.7	4.7	7.2	7.0	12.0	11.3	8.9	8.5	16.1	15.1
19.8	4.4	4.3	4.7	4.7	7.3	7.1	12.0	11.4	9.0	8.6	16.2	15.1
19.8	4.4	4.3	4.7	4.8	7.3	7.1	12.1	11.4	9.0	8.6	16.2	15.2
19.9	4.5	4.4	4.8	4.8	7.4	7.2	12.1	11.5	9.1	8.7	16.3	15.2

Figures

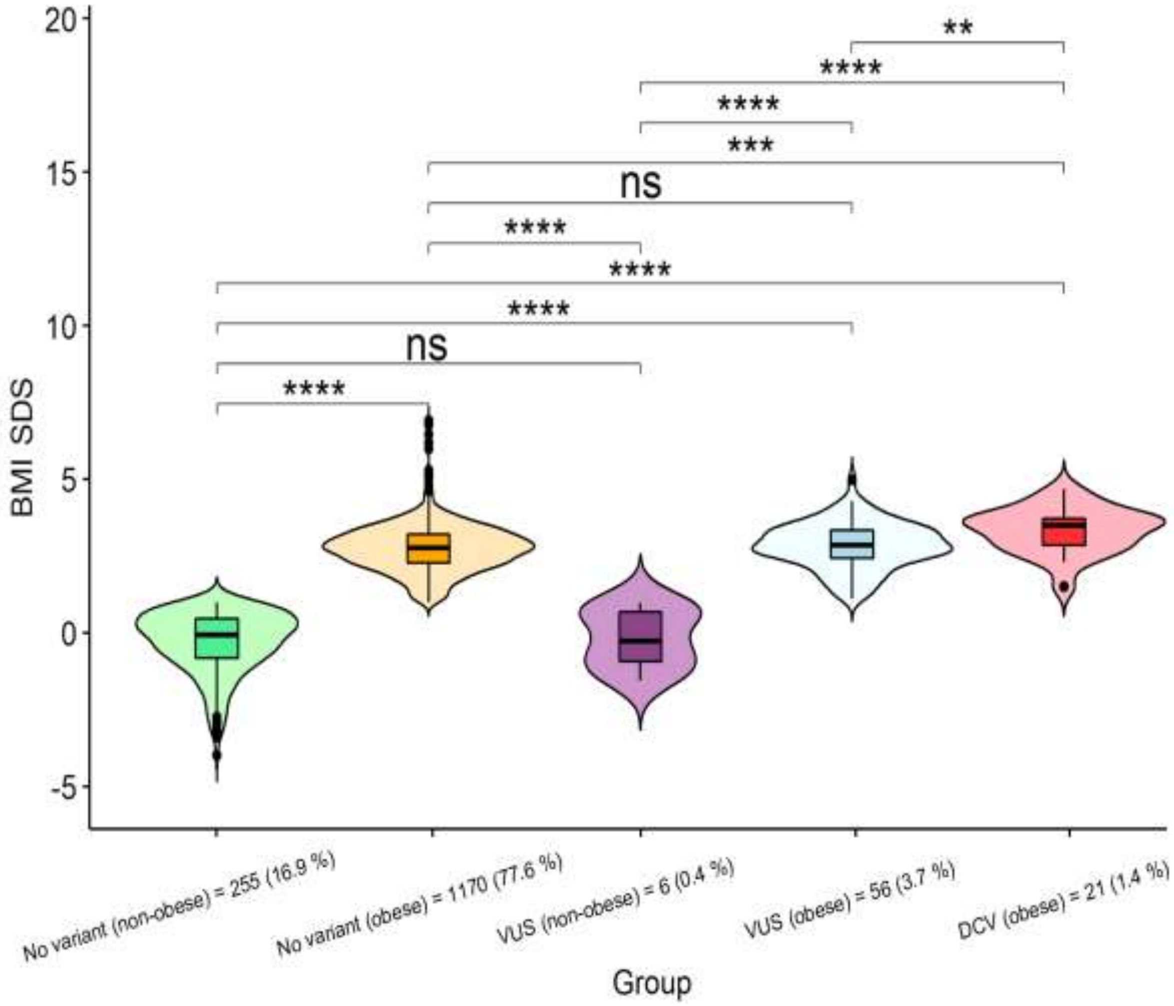

Figure S1: Significant difference in BMI SDS between: (i) participants without obesity and without identified variant (green), (ii) participants with obesity and without identified variant (orange), (iii) participants without obesity and without VUS (purple), (iv) participants without obesity and with VUS (blue), and (v) participants without obesity and with a confirmed disease-causing variant (red). P-value legend: P > 0.05 (ns); P ≤ 0.05 (*); P ≤ 0.01 (**); P ≤ 0.001 (***); P ≤ 0.0001(****).

Text S1: Bioinformatic pipeline. The generated data was filtered and aligned to the reference genome GRCh37 (hg19) using BWA aligner (Li and Durbin, 2009). After marking of duplicates and base quality score recalibration using the Genome Analysis Toolkit (GATK) (Mckenna et al., 2010), variant calling was performed using an ensemble of algorithms GATK-HaplotypeCaller (Mckenna et al., 2010), VarDict (Lai et al., 2016), Strelka2 (Saunders et al., 2012) and FreeBayes (Garrison and Marth, 2012). Using BEDTools (Quinlan and Hall, 2010) required coverage of the targeted gene's nucleotides was > 50 x, with mapping quality score > 30. Detected variations in genomic data (single-nucleotide variants (SNVs) and short insertions/deletions (indels)) were functionally annotated and filtered for rare variants (MAF < 1%) using the VarAFT tool (Desvignes et al., 2018), databases, i.e. 1000 Genomes (Auton et al., 2015), gnomAD (Karczewski et al., 2020), ClinVar (Landrum et al., 2014), and deleteriousness predictors, i.e. SIFT (Ng and Henikoff, 2003), PolyPhen-2 (Adzhubei et al.,

2010), MutationTaster (Schwarz et al., 2010), MutationAssessor (Reva et al., 2011), FATHMM (Shihab et al., 2013), and CADD (Rentzsch et al., 2019).

Literature

Adzhubei, I. A., Schmidt, S., Peshkin, L., Ramensky, V. E., Gerasimova, A., Bork, P., et al. (2010). A method and server for predicting damaging missense mutations. *Nat. Methods* 7, 248–249. doi:10.1038/nmeth0410-248.

Auton, A., Brooks, L., and Durbin, R. (2015). A global reference for human genetic variation. *Nature* 526, 68–74. doi:10.1038/nature15393.

de Onis, M., Onyango, A., Borghi, E., Siyam, A., Blössner, M., Lutter, C., et al. (2012). Worldwide implementation of the WHO Child Growth Standards. *Public Health Nutr.* 15, 1603–1610. doi:10.1017/S136898001200105X.

Desvignes, J., Bartoli, M., Krahn, M., Miltgen, M., Christophe, B., and Salgado, D. (2018). VarAFT : a variant annotation and filtration system for human next generation sequencing data. *Nucleic Acids Res.* 46, W545–W553. doi:10.1093/nar/gky471.

Flegal, K. M., and Cole, T. J. (2013). Construction of LMS parameters for the Centers for Disease Control and Prevention 2000 growth charts. *Natl. Health Stat. Report.*, 1–4.

Garrison, E., and Marth, G. (2012). Haplotype-based variant detection from short-read sequencing. Preprint at https://arxiv.org/abs/1207.3907.

Karczewski, K. J., Francioli, L. C., Tiao, G., Cummings, B. B., Alföldi, J., Wang, Q., et al. (2020). The mutational constraint spectrum quantified from variation in 141 , 456 humans. *Nature* 581, 434–443. doi:10.1038/s41586-020-2308-7.

Lai, Z., Markovets, A., Ahdesmaki, M., Chapman, B., Hofmann, O., Mcewen, R., et al. (2016). VarDict : a novel and versatile variant caller for next-generation sequencing in cancer research. *Nucleic Acids Res.* 44, e108. doi:10.1093/nar/gkw227.

Landrum, M. J., Lee, J. M., Riley, G. R., Jang, W., Rubinstein, S., Church, D. M., et al. (2014). ClinVar : public archive of relationships among sequence variation and human phenotype. *Nucleic Acids Res.* 42, D980–D985. doi:10.1093/nar/gkt1113.

Li, H., and Durbin, R. (2009). Fast and accurate short read alignment with Burrows – Wheeler transform. *Bioinformatics* 25, 1754–1760. doi:10.1093/bioinformatics/btp324.

Mckenna, A., Hanna, M., Banks, E., Sivachenko, A., Cibulskis, K., Kernytsky, A., et al. (2010). The Genome Analysis Toolkit : A MapReduce framework for analyzing next-generation DNA sequencing data. *Genome Res.* 20, 1297–1303. doi:10.1101/gr.107524.110.20.

Ng, P. C., and Henikoff, S. (2003). SIFT : predicting amino acid changes that affect protein function. *Nucleic Acids Res.* 31, 3812–3814. doi:10.1093/nar/gkg509.

Quinlan, A. R., and Hall, I. M. (2010). BEDTools : a flexible suite of utilities for comparing genomic

features. *Bioinformatics* 26, 841–842. doi:10.1093/bioinformatics/btq033.

Rentzsch, P., Witten, D., Cooper, G. M., Kircher, M., and Shendure, J. (2019). CADD : predicting the deleteriousness of variants throughout the human genome. *Nucleic Acids Res.* 47, D886–D894. doi:10.1093/nar/gky1016.

Reva, B., Antipin, Y., and Sander, C. (2011). Predicting the functional impact of protein mutations : application to cancer genomics. *Nucleic Acids Res.* 39, e118. doi:10.1093/nar/gkr407.

Richards, S., Aziz, N., Bale, S., Bick, D., Das, S., Gastier-Foster, J., et al. (2015). Standards and guidelines for the interpretation of sequence variants: A joint consensus recommendation of the American College of Medical Genetics and Genomics and the Association for Molecular Pathology. *Genet. Med.* 17, 405–424. doi:10.1038/gim.2015.30.

Saunders, C. T., Wong, W., Swamy, S., Becq, J., Murray, L. J., and Cheetham, R. K. (2012). Strelka : Accurate somatic small-variant calling from sequenced tumor-normal sample pairs . *Bioinformatics* 28, 1811–1817.

Schwarz, J. M., Rödelsperger, C., Schuelke, M., and Seelow, D. (2010). MutationTaster evaluates disease-causing potential of sequence alterations. *Nat. Methods* 7, 575–576. doi:10.1038/nmeth0810-575.

Shihab, H. A., Gough, J., Cooper, D. N., Stenson, P. D., Barker, G. L. A., Edwards, K. J., et al. (2013). Predicting the functional, molecular, and phenotypic consequences of amino acid substitutions using hidden Markov models. *Hum. Mutat.* 34, 57–65. doi:10.1002/humu.22225.

World Health Organization (2019). WHO Global Database on Child Growth and Malnutrition. Available at: http://www.who.int/nutgrowthdb [Accessed December 4, 2019].
